# Effects of Seasonal Changes on Chlorophyll Fluorescence and Physiological Characteristics in the Two *Taxus* Species

**DOI:** 10.3390/plants12142636

**Published:** 2023-07-13

**Authors:** Tao Wang, Lingyu Li, Yalong Qin, Bo Lu, Donghuan Xu, Weibing Zhuang, Xiaochun Shu, Fengjiao Zhang, Ning Wang, Zhong Wang

**Affiliations:** 1 Jiangsu Key Laboratory for the Research and Utilization of Plant Resources, Institute of Botany, Jiangsu Province and Chinese Academy of Sciences (Nanjing Botanical Garden Mem. Sun Yat-Sen), Nanjing 210014, China; johnwt1007@163.com (T.W.); xu15689774892@163.com (D.X.); weibingzhuang@cnbg.net (W.Z.);; 2 Nanjing Athortiland Agricultural Science and Technology Development Co., Ltd., Nanjing 210043, China; 15366105463@163.com

**Keywords:** chlorophyll *a* transient, chlorophyll fluorescence imaging, photosynthetic response, seasonal dynamics, *Taxus* species

## Abstract

*Taxus* is a rare and endangered woody plant worldwide with important economic and ecological values. However, the weak environmental adaptability of *Taxus* species, in particular the unstable photosynthetic activity in different seasons, always affects its normal growth and development and limits its conservation and exploitation. To improve the survival of *Taxus* trees in cultivated areas, the seasonal dynamics of chlorophyll fluorescence (CF) and key physiological parameters were comprehensively investigated in *T. media* and *T. mairei*. The results demonstrated that the photosynthetic activity of both *Taxus* species was sensitive to local summer and winter environmental conditions, with the heterogeneity of fluorescence signatures intuitively presented on the needle surface by CF-Imaging detection, while images of maximum quantum efficiency of PSII photochemistry (Fv/Fm) demonstrated values below 0.7 in the blue–green sectors in winter. The distribution of light energy was regulated by the photosynthetic apparatus in both *Taxus* species to maintain a stable actual quantum yield of PSII photochemistry (φPSII), which was around 0.4–0.5. Based on a redundancy discriminant analysis, the interpretation rate of light intensity and air temperature ranked as the top two in both *Taxus* species, which were considered the main environmental factors affecting the photosynthetic performance of *Taxus* by disturbing the electron transport chain. In the winter, *T. mairei* exhibited weaker electron transport activity than *T. media*, thus caused lower photochemistry and more severe photosynthetic damages. Interestingly, both *Taxus* species demonstrated consistent response patterns, including diverse energy dissipation strategies and enhancement of osmoregulatory substances and antioxidative activities, thus maintaining stable photosynthetic functions in response to environmental changes.

## 1. Introduction

*Taxus* is a small coniferous tree or shrub, which belongs to the genus *Taxus* and family Taxaceae, with great medicinal, ornamental, and ecological values. The *Taxus* species is well-known for the important biological active compound taxol, an effective drug used in the treatment of various cancers such as lung, breast, and ovarian [1,2]. However, the content of taxol in *Taxus* species is extremely low; only 1 kg of taxol could be extracted from thousand-century-old *Taxus* trees [3]. As the main and reliable source for taxol and its precursors, the *Taxus* species has almost suffered extinction due to over-exploitation and illegal trade, while all the members of the genus *Taxus* have been listed as species for first-grade protection in the National Key Protected Wild Plants List in China [4]. Therefore, it is urgent to explore a mature system for the protection of *Taxus* from endangerment in the alleviation of the market supply crisis.

The latest literature has reported that the genus *Taxus* is composed of thirteen recognized species plus three additional cryptic species, which are loosely distributed across temperate regions of the northern hemisphere, including North America, Europe, North Africa, and Asia [5]. *Taxus* grows slowly; together with a poor reproductive capacity and competitiveness in populations, it rarely occurs in large numbers [6]. Moreover, long-term geographical isolation also causes great differences in environmental adaptability within different species. For example, *T. media* could tolerate extreme cold temperatures around −20 °C [7]; such a low temperature was considered a great limiting factor for the growth and distribution of *T. mairei* [8]. The limited resources call for a study on the growth and development of the *Taxus* species and its response to environmental changes, which is conducive to more scientific and efficient cultivation of *Taxus*.

Photosynthesis is one of the most sensitive components in response to environmental changes [9,10,11]. Previous studies have reported the photosynthetic capacity of *Taxus* species under different environmental conditions, such as temperature [12], water [13], light [14,15,16], and soil [17,18]. However, most of the reports were obtained by the measurements under the control of a single environmental factor for a single *Taxus* species in the short or medium term; while less focused on the response characteristics under long seasonal alternations or among different species. As a typical evergreen tree, the *Taxus* shows seasonal changes in phenology, which inevitably causes differences of photosynthetic characteristic parameters. The corresponding response strategies are also important clues for the succession and development of the *Taxus* community. Therefore, it is necessary to investigate the seasonal response characteristics of photosynthesis in *Taxus*, which is crucial for a comprehensive elaboration of the photosynthetic response mechanism.

Notably, almost all changes in photosynthetic processes could be reflected by chlorophyll (Chl) fluorescence kinetics [19]. The changes in photosystem II (PSII) photochemistry prior to/after dark reaction activation can be obtained by a continuous excitation fluorometer [20,21] and a pulse-modulated fluorometer [22], respectively. Recently, the Chl fluorescence imaging (CF-Imaging) technology was used to monitor and diagnose the responses of plant photosynthesis to environmental stresses, such as temperature [23,24], water [25], salt [26], and light [27,28], and could be a useful method for the screening of stress-tolerant or -sensitive genotypes, as it could simultaneously obtain the Chl fluorescence parameters and spatial heterogeneity distribution in plant leaves [29,30]. The combination of multiple Chl fluorescence methods for seasonal observations will provide a comprehensive understanding of primary photochemical reactions in *Taxus* species.

Moreover, photosynthesis is also the main source of reactive oxygen species (ROS) [31], which were recognized as toxic by-products of aerobic metabolism [32]. Therefore, ROS scavenging systems are required in photosynthetic processes to balance ROS pools and alleviate potential oxidative damages. Superoxide dismutase (SOD) and Peroxidase (POD) are two main antioxidant enzymes, which are considered as sensors to prevent the occurrence of oxidative stress [8]. The primary product of oxygen reduction superoxide anion is disproportionated by SOD to molecular oxygen and hydrogen peroxide (H_2_O_2_); while the latter is subsequently reduced by POD to water with the generation of monodehydroascorbate. Interestingly, the antioxidants not only have specific protective functions, but also demonstrate associations with a trade-off between survival and reproduction [17]. Higher levels of antioxidants tend to higher metabolic activity; while increased metabolism leads to higher production of ROS, which in turn disrupts protein stability and carbohydrate synthesis. Therefore, a highly activated antioxidant system is vital for the maintenance of photosynthetic homeostasis [33,34].

*T. media*, a hybrid of *T. baccata* × *T. cuspidata*, has been cultivated in the United States and Canada for over 100 years with high taxol content and strong renewable capacity [35]. *T. mairei* is endemic to China with good environmental adaptability and wide distribution [36]. Since the 1980s, these two *Taxus* species have been introduced into Nanjing Botanical Garden Mem. Sun Yat-Sen for cultivation and targeted research. In such conditions, the comparison of the photosynthetic responses to seasonal changes in *Taxus* species from different geographical sources could be conducted smoothly. It was predicted that these two *Taxus* species could possess different photosynthetic response strategies and protection mechanisms for the photosynthetic apparatus in different seasons based on the geographical distribution pattern of their wild resources and many years of local phenological observations. In this study, these two representatives were selected for dynamic measurements of Chl fluorescence and key physiological parameters in different months. The aim was to investigate the seasonal differences in photosynthetic and physiological characteristics, explore the specificity among different *Taxus* species and confirm the main environmental limiting factors at different time scales, thus comprehensively elaborate the process and related mechanism of photosynthetic physiological responses for *Taxus*. The results of this study will provide a reference for the assessment of survival states and rational conservation and application of *Taxus* species.

## 2. Results

### 2.1. Seasonal Dynamics of Chlorophyll Fluorescence Parameters and Images in T. media and T. mairei

As shown in Table 1, the Fv/Fm of *T. media* and *T. mairei* demonstrated a fluctuating upward trend from January to April, and remained at 0.77–0.83 from April to September, then gradually decreased. The lowest values in both *Taxus* species appeared in January, around 0.60–0.70. Significant differences of Fv/Fm values between the two *Taxus* species were observed in January-March and June. The Fv′/Fm′ showed similar trends as Fv/Fm from January to April, then maintained relatively stable levels until December. The values of *T. media* in March, May, June, August, and November were significantly higher than that of *T. mairei*. The NPQ of *T. mairei* increased from January to April, and remained above 2 from April to November (except for June), but dropped to 1.35 in December; while that of *T. media* fluctuated during the measurements, and was below 2 after July. The lowest NPQ values of *T. media* and *T. mairei* were observed in January around 1.22 and 0.88, respectively. The trends of qP in *Taxus* species were opposite to those of Fv′/Fm′. The highest qP values of *T. media* and *T. mairei* appeared in January, around 0.86 and 0.96, respectively. The trends of φPSII were relatively stable throughout the annual observation. The φPSII values of *T. media* were higher than that of *T. mairei* after February, and significant differences were observed in March, July, and August.

**Figure 1:**
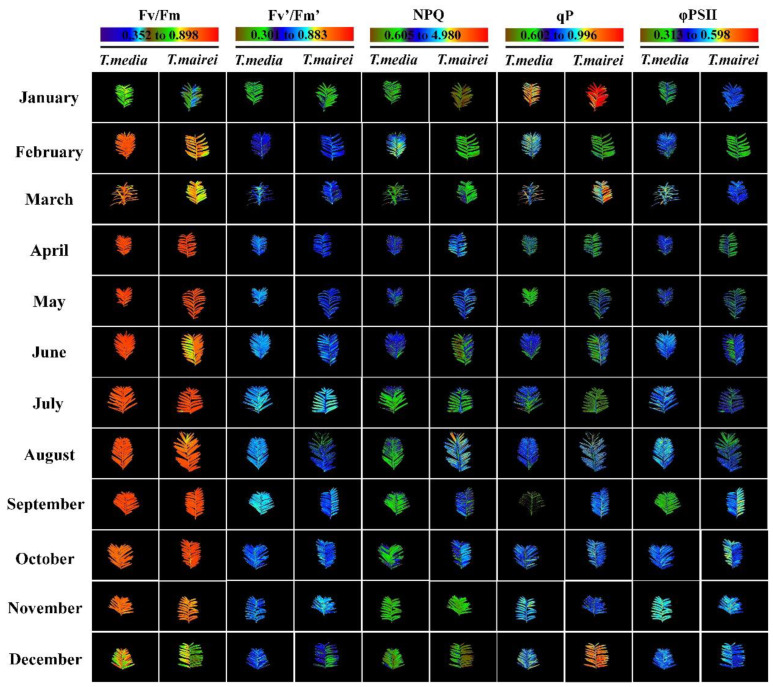
False color images of fluorescence parameters in *T. media* and *T. mairei* leaves from January to December.

Figure 1 shows representative false color images of Fv/Fm, Fv′/Fm′, NPQ, qP, and φPSII by CF-Imaging, which visually represented the spatial differences over the leaf surface of *T. media* and *T. mairei* in different months. Among them, the images of Fv/Fm exposed significant heterogeneity. In most cases, the leaves of both *Taxus* species were an orange-red color, which turned blue-green in January, and yellow-orange in February and March, as well as green-yellow after October, along with decreasing Fv/Fm values in varying degrees. Moreover, the leaves of *T. mairei* were heterogeneous within orange sectors between June and August, which also affected the comprehensive values. The images of Fv′/Fm′ and φPSII in the two *Taxus* species showed significant differences between the green and blue-cyan sectors. The leaves in most cases showed blue-cyan colors, which represented relatively high levels as compared with the green ones observed in the Fv′/Fm′ of both *Taxus* species in January, as well as φPSII of *T. mairei* in February and that of *T. media* in August. This was confirmed by the numeric data. The qP images resembled that of Fv′/Fm′ and φPSII, revealing a heterogeneity of this parameter within the blue-cyan area. Moreover, orange-red sectors were also observed in the leaves of *Taxus* species within several months, which showed higher levels than the blue-cyan parts. The NPQ in *T. media* leaves was lower than in *T. mairei* ones after July, and lower in the green parts than in the blue-cyan areas. This parameter was also low in the brown parts as compared with the green ones, which could be observed in January and December. Notably, the heterogeneity of Chl fluorescence occurred not only on the whole plant leaves, but also on part of the whole plant leaves or parts of the leaves, such as leaf margins and veins, which determined the degree of changes in comprehensive values of Chl fluorescence parameters. This was particularly evident for Fv/Fm and φPSII, which also reflected the complexity of the photosynthetic response.

### 2.2. Changes of Chlorophyll a Fluorescence Transient and JIP-Test Parameters in T. media and T. mairei

**Figure 2:**
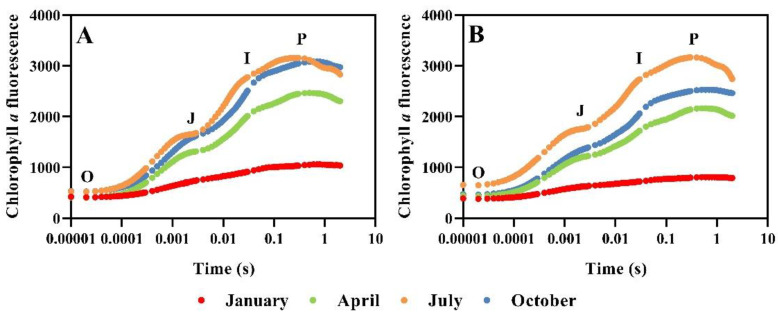
Chlorophyll *a* fluorescence transient induction curves of *T. media* (**A**) and *T. mairei* (**B**) in January, April, July, and October. Data are shown as the average of 4 replicates.

Given the sensitivity of primary photochemical reactions to environmental changes in *Taxus* species, the Chl *a* fluorescence transient has been applied to monitor changes in the structure and states of the photosynthetic apparatus in representative months. As shown in Figure 2, the polyphasic OJIP rise was observed in leaves of *Taxus* species under different months. Notably, the J-I and I-P phases of both *Taxus* species increased significantly in July compared with that in April; while same phases of *T. media* were also higher in October than that in April. Moreover, the fluorescence intensity of OJIP rise in January decreased significantly and caused changes in the curve shapes. There was no obvious K-band appearing at 0.3 ms; however, the curves became flat as the J-, I-, and P-steps decreased sharply, which exhibited the same pattern in both *Taxus* species.

**Figure 3:**
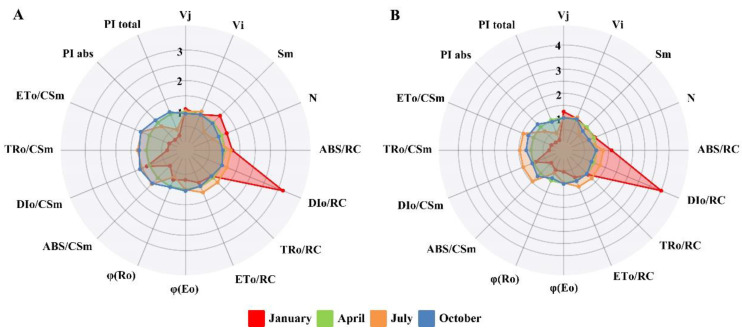
Spider plots of selected chlorophyll *a* fluorescence parameters of *T. media* (**A**) and *T. mairei* (**B**) in January, April, July, and October. Data are shown as the average of 4 replicates.

To further evaluate the condition of the photosynthetic apparatus in *Taxus* species, selected parameters were calculated from JIP-test (Table S2) and were normalized for spider plots with values measured in April used as the respective control (Figure 3). In this study, multiple parameters of both *Taxus* species exhibited significant changes in January and July as compared with that in April. Among them, Vj and Vi of *T. mairei* in January increased by 24.09% and 6.66%; meanwhile, Sm of *T. media* increased by 31.67%. Vi of both *Taxus* species increased in July, which was opposite to their trends of Sm and N. The φEo of *T. media* and *T. mairei* only decreased by 31.09% and 46.84% in January; while their φR_0_ decreased significantly in January and July, respectively. Notably, both *Taxus* species demonstrated increased ABS/RC and DI_0_/RC in January; however, their ABS/CSm decreased sharply in January, similar to the trends of TR_0_/CSm and ET_0_/CSm. In contrast, ABS/RC, TR_0_/RC, and ET_0_/RC of both *Taxus* species in July were higher than those in April, which were consistent with their corresponding parameters per CS. Moreover, DI_0_/CSm of *T. media* and *T. mairei* also increased by 25.93% and 45.74% in July. Apparently, both *Taxus* species demonstrated lower PI_abs_ and PI_total_ values in January and July than in April, with the former decreased more sharply.

### 2.3. Seasonal Dynamics of Chlorophyll and Carotenoid Contents in T. media and T. mairei

**Figure 4:**
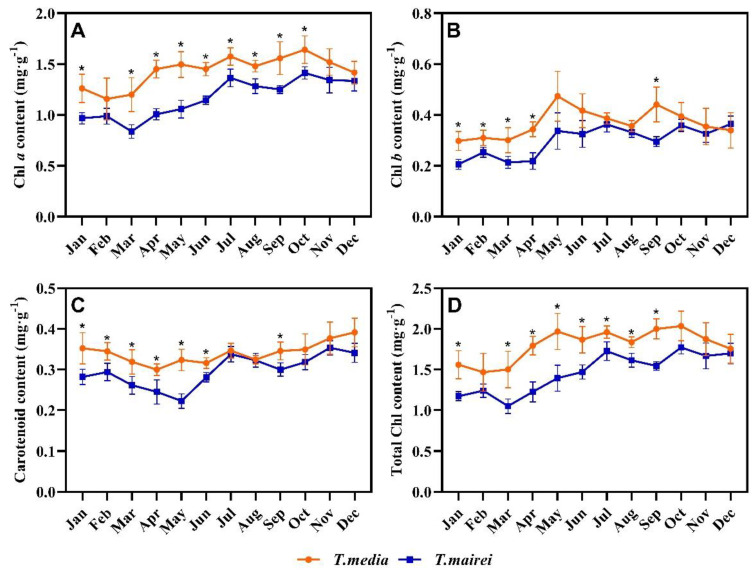
Seasonal dynamics of chlorophyll *a* (**A**), chlorophyll *b* (**B**), carotenoid (**C**) and total chlorophyll (**D**) content in *T. media* and *T. mairei.* Data are shown as mean ± SE (*n* = 3). Asterisk indicates significant difference between two *Taxus* species in the same month according to Student’s *t*-test (*p* < 0.05).

As shown in Figure 4, the Chl and carotenoid contents in *T. media* and *T. mairei* fluctuated in different months. For Chl *a*, the lower levels in both *Taxus* species were observed between January and March; while the highest was observed in October, around 1.41 and 1.64 mg g^−1^, respectively. The Chl *a* content of *T. media* was higher than that of *T. mairei* in different months, and significant differences were observed in January and March–October (Figure 4A). The Chl *b* content in *T. media* demonstrated a higher accumulation in May and September, with values around 0.47 and 0.44 mg g^−1^, respectively; meanwhile, the peak in *T. mairei* was 0.36 mg g^−1^ in July. The lowest level of Chl *b* in both *Taxus* species was observed in January, which was 0.21 and 0.30 mg g^−1^, respectively. Significant differences for Chl *b* values between *T. media* and *T. mairei* were demonstrated in January–April and October (Figure 4B). The carotenoid content in *T. media* varied gently in different months, with a maximum of 0.39 mg g^−1^ in December; meanwhile, that in *T. mairei* demonstrated the lowest in May at 0.22 mg g^−1^, and reached the highest at 0.35 mg g^−1^ in November with a strong fluctuating rise. Significant differences between *T. media* and *T. mairei* were in January–June and September (Figure 4C). The trend of total Chl content in *Taxus* species was similar with that of Chl *a* content. The lowest content in *T. media* and *T. mairei* was observed in February and March, respectively, with values around 1.47 and 1.05 mg g^−1^; meanwhile, the highest was obtained in October, which was 1.77 and 2.04 mg g^−1^. The total Chl content of *T. media* was significantly higher than that of *T. mairei* in January and March–September (Figure 4D).

### 2.4. Seasonal Dynamics of Soluble Protein and Soluble Sugar Content in T. media and T. mairei

**Figure 5:**
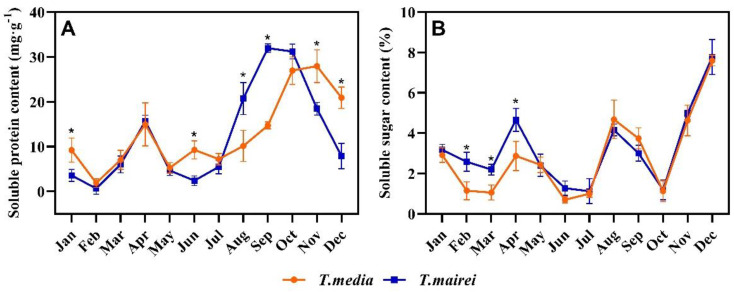
Seasonal dynamics of soluble protein (**A**) and soluble sugar (**B**) content in *T. mairei* and *T. media.* Data are shown as mean ± SE (*n* = 3). Asterisk indicates significant difference between two *Taxus* species in the same month according to Student’s *t*-test (*p* < 0.05).

The soluble protein content in *T. media* and *T. mairei* demonstrated a fluctuating trend from January to May with a similar accumulation (except for January); after June, that of both increased strongly at first and then decreased rapidly, but the responses of *T. mairei* to environmental changes were earlier than that of *T. media*. The lowest value in both *Taxus* species was observed in February, around 0.65 and 1.88 mg g^−1^, respectively; while the highest was 31.95 mg g^−1^ in September for *T. mairei* and was 27.93 mg g^−1^ in November for *T. media* (Figure 5A). The soluble sugar content in two *Taxus* species demonstrated similar trends as their soluble protein from January to April, with values in *T. mairei* being significantly higher than that in *T. media* (except for January). Then, both *Taxus* species demonstrated fluctuating trends, but with insignificant difference of values within. The lowest value in *T. mairei* was at 1.12% in July; while that in *T. media* was at 0.70% in June. The highest value in both *Taxus* species appeared in December, which was at 7.77% and 7.60%, respectively (Figure 5B).

### 2.5. Seasonal Dynamics of SOD and POD Activity in T. media and T. mairei

**Figure 6:**
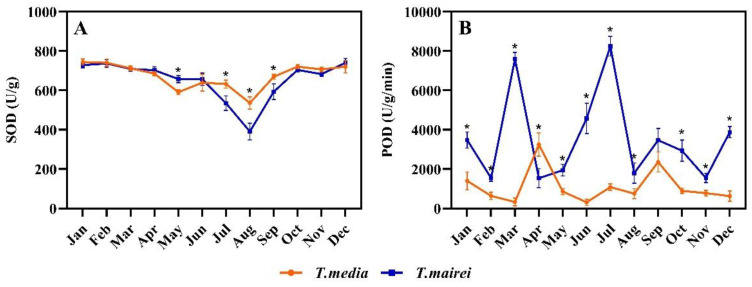
Seasonal dynamics of SOD (**A**), POD (**B**) activity in *T. mairei* and *T. media.* Data are shown as mean ± SE (*n* = 3). Asterisk indicates significant difference between two *Taxus* species in the same month according to Student’s *t*-test (*p* < 0.05).

The SOD activity of both *Taxus* species maintained relatively high levels with an insignificant difference from January to April as well as from October to December. In May, the SOD activity of *T. mairei* was 11.27% higher than that of *T. media* with a significant difference; from July to September, that of both demonstrated a trend of decreasing first then increasing, and the values in *T. media* were significantly higher than that in *T. mairei*. The lowest value in both *Taxus* species appeared in August, which was 391.20 and 536.11 U/g, respectively (Figure 6A). The POD activity of *T. mairei* demonstrated an obvious fluctuating trend during the measurements with two extremely high peaks occurred in March and July, respectively. The trend of POD activity in *T. media* was similar with that in *T. mairei*, but the first peak appeared in April, whose responses to environmental changes were relatively late; while the second peak was less pronounced. Notably, the values of *T. mairei* in most months were significantly higher than that of *T. media* (Figure 6B).

### 2.6. Pearson Correlation Analysis of Chl Fluorescence and Physiological Indicators in T. media and T. mairei

**Figure 7:**
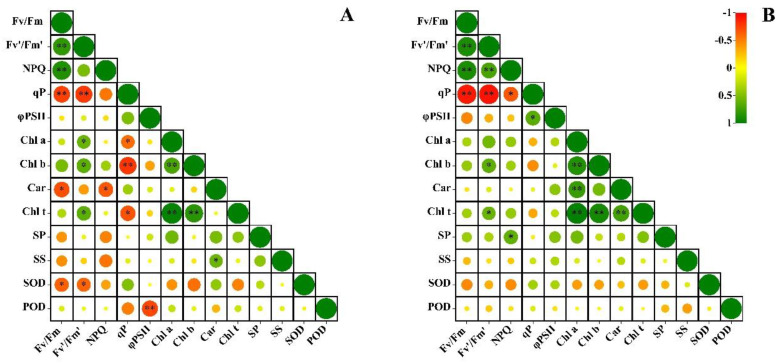
Pearson correlation analysis of chlorophyll fluorescence and physiological indicators in *T. media* (**A**) and *T. mairei* (**B**). Shown are the heatmap of Pearson correlation coefficient among chlorophyll fluorescence and physiological indicators in *Taxus* species. * indicates significant correlations at *p* < 0.05 level; ** indicates significant correlations at *p* < 0.01 level. Car, carotenoid; Chl *a*, chlorophyll *a*; Chl *b*, chlorophyll *b*; Chl t, the total chlorophyll; SP, soluble protein; SS, soluble sugar.

The Pearson correlation analysis was performed to evaluate the relationships among the Chl fluorescence and physiological indicators in the two *Taxus* species (Figure 7). In *T. media*, the Fv/Fm was highly positively correlated with Fv′/Fm′ and NPQ, and highly negatively correlated with qP (*p* < 0.01), as was the correlation between Fv′/Fm′ and qP (*p* < 0.01). Moreover, the Fv′/Fm′ was positively correlated with Chl *a*, Chl *b*, and the total Chl content, respectively (*p* < 0.05); meanwhile, the qP was highly negatively correlated with Chl *b* (*p* < 0.01), and negatively correlated with Chl *a* and the total Chl content (*p* < 0.05). There was also a highly positive correlation in the Chl *a*, Chl *b*, and total Chl content, and a highly negative correlation between φPSII and POD activity (*p* < 0.01). Among other indicators, the SOD activity was negatively correlated with Fv/Fm and Fv′/Fm′ (*p* < 0.05); the carotenoid content was negatively correlated with Fv/Fm and NPQ, and positively correlated with soluble sugar content (*p* < 0.05). The correlation trend of Chl fluorescence parameters in *T. mairei* was basically the same as that in *T. media*; meanwhile, the NPQ in *T. mairei* was additionally highly positively correlated with Fv′/Fm′ (*p* < 0.01) and negatively correlated with qP (*p* < 0.05). Interestingly, *T. mairei* demonstrated highly positive correlations in photosynthetic pigment content (*p* < 0.01), except between Chl *b* and carotenoid content. Moreover, there were positive correlations between Fv′/Fm′ and Chl *b* or the total Chl content as well as the NPQ and soluble protein content in *T. mairei* (*p* < 0.05).

### 2.7. Redundancy Discriminant Analysis between Environmental Factors and Chlorophyll Fluorescence and Physiological Indicators in T. media and T. mairei

**Figure 8:**
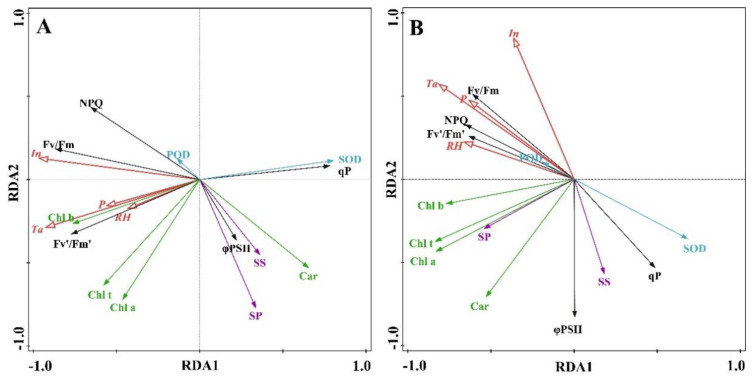
Redundancy discriminant analysis of environmental factors and chlorophyll fluorescence and physiological indicators in *T. media* (**A**) and *T. mairei* (**B**). The red line indicates environmental factors, while other line with different colors indicates Chl fluorescence and physiological indicators. Car, carotenoid; Chl *a*, chlorophyll *a*; Chl *b*, chlorophyll *b*; Chl t, the total chlorophyll; SP, soluble protein; SS, soluble sugar.

The effects of different environmental factors on Chl fluorescence and physiological characteristics in the two *Taxus* species were analyzed by RDA (Figure 8). The representative environmental factors were clearly distributed on the left side of the RDA1 axis in both *Taxus* species. The interpretation rate in *T. media* was ranked as In > Ta > RH > P; while that in *T. mairei* was ranked as Ta > In > RH > P. Among them, In, Ta, and RH in both *Taxus* species significantly affected Chl fluorescence and physiological parameters (*p* < 0.1). For *T. media* and *T. mairei*, the environmental factors were closely related to Chl fluorescence indicators and ROS scavenging enzymes. According to the principle of RDA, the included angle between environmental factors and Chl fluorescence and physiological indicators is acute, indicating that they are positively correlated, while obtuse angles indicate negative correlations. Therefore, the SOD activity, qP, and φPSII were negatively correlated with environmental factors, while other indicators were positively correlated with environmental factors.

## 3. Discussions

The advantage of CF-Imaging technology was permitting the study of photosynthetic activity over the entire leaf surface. Through the false color palettes, where different colors encode for different values of Chl fluorescence parameters, the spatial and temporal heterogeneity of Chl fluorescence signatures could be represented pixel by pixel in the images [25,30]. In this study, CF-Imaging provides intuitive and accurate information on the seasonal responses of photosynthetic performance in *Taxus* leaves. Among them, images of Fv/Fm in *Taxus* demonstrated healthy orange-red colors throughout the entire leaf area in spring and autumn; meanwhile, the heterogeneity was mainly represented as green-yellow and blue-green sectors in summer and winter. The Fv/Fm is a reliable indicator to detect some kinds of early stresses in plants, such as high irradiance and low temperature, which persists in the normal range of 0.8 or so under suitable environmental conditions, and always decreases due to the changes in the use of light energy and photoinactivation of PSII RCs [37,38]. However, Epron et al. [39] also reported that Fv/Fm could be unaffected under drought stress until the complete cessation of CO_2_ assimilation. The changes of Fv/Fm in this study indicated that the photosynthetic apparatus of *Taxus* differed in sensitivity to different seasons and was partially or fully susceptible in summer and winter environments. This also implied that water deficit was not the main factor affecting the photosynthetic performance of *Taxus* during seasonal changes. Moreover, images of other Chl fluorescence parameters also demonstrated a distribution of heterogeneity with different visualization effects during the measurements; but their accuracy or sensitivity is less than that of Fv/Fm. According to the results of the evaluation of all Chl fluorescence parameters, the Fv/Fm-based CF-Imaging has the potential to be developed as a simple and effective tool for assessing the physiological stress states of *Taxus*.

Literatures have reported that the Chl and carotenoid contents were the basis for the interpretation of Chl fluorescence parameters and the origin of multicolor fluorescence imaging [40,41,42]. Therefore, the Chl and carotenoid contents of the two *Taxus* species in different months could provide a reasonable explanation for their photochemistry and image heterogeneity. Overall, the Chl contents of *Taxus* demonstrated significant positive correlations with Fv′/Fm′, and negative correlations with qP. This suggested that light energy utilization for photochemistry in *Taxus* leaves could be improved by controlling the number of functioning PSII RCs under light conditions [43]. Interestingly, qP of both *Taxus* species increased to varying degrees in winter, which was the opposite of NPQ. Combined with the analysis of other Chl fluorescence parameters, it suggested a low level of PSII, presumably as a result of energy migration occurring between PSII and PSI, based on the contribution of Chl and carotenoids to form aggregates of photosystem complexes [44]. Carotenoids involved in photosynthesis are bound to the light-harvesting pigment-protein complexes of photosystems [45]. The significant correlations between carotenoid and Chls as well as between carotenoid and Chl fluorescence parameters were respectively observed in *T. mairei* and *T. media*, which further emphasized the crucial roles of carotenoids on the light harvesting, energy transfer and photoprotection in these two *Taxus* species [46]. All the efforts were aimed at maintaining a relatively consistent φPSII, which also benefits from the feedback regulation of photosynthetic carbon assimilation [22]. This could be an important mechanism for photochemical responses of *Taxus* to different environmental conditions, and should be considered for the detection of injury degree.

The Chl *a* fluorescence transient is characterized by a polyphasic fluorescence increase, which reflects the information on the O-P phases of Chl fluorescence kinetics [47]. The JIP-test analysis, based on the so-called “Theory of Energy Fluxes in Biomembranes”, involves translating the fluorescence transients into several phenomenological and biophysical expressions [48,49]. In this study, a series of basic fluorescence parameters involved in specific energy fluxes, phenomenological energy fluxes, quantum efficiencies, and a performance index were selected to further study the photosynthetic responses of *Taxus* to seasonal changes. For both *Taxus* species, the specific energy fluxes (ABS/RC, TR_0_/RC, and ET_0_/RC) increased significantly in July compared with that in April; while the corresponding phenomenological energy fluxes (ABS/CSm, TR_0_/CSm, and ET_0_/CSm) also demonstrated a consistent trend. This suggested that the absorption and conversion of light energy in *Taxus* were enhanced in the summer environment. However, the decrease in Sm and N indicated a decreased pool size of electron carriers, which potentially affected the reduction events of Q_A_ [50]. Moreover, the increased Vi further implied diminished electrons transport to Q_B_, which ultimately affected the reduction at the PSI acceptor side (φR_0_). This situation was basically consistent with the theory term as “energy trapping” [22], which was considered a specific mechanism to protect the photosynthetic apparatus from potential environmental stresses. However, it simultaneously sacrificed the photosynthetic performance, which was reflected by a significant decrease in the performance indexes (PI_total_ and PI_abs_). Moreover, the φE_0_ and φR_0_ of both *Taxus* species were lower in January than that in April, indicating a weak activity of the electron transport chain; while the increased ABS/RC and DI_0_/RC represented a strong absorption and dissipation of light energy at the level of the antenna Chl. Interestingly, the phenomenological energy fluxes decreased sharply, which could be the result of a decrease in the opening ratio of active RCs. These combined results further decreased the performance indexes compared with that in July; meanwhile, the difference of the parameters above in the two *Taxus* species could reasonably explain their different photochemistry. Fortunately, this situation demonstrated a recovery in April, which further implied that the *Taxus* has the potential of a reversible deactivation for RCs in winter. It appears that the continuous and diverse energy dissipation mechanism is the key to the survival of evergreen conifers, such as *Taxus* [51].

The RDA is a two-table method in which the gradient found in the Chl fluorescence and physiological parameters could be directly related to the external environmental factors [52]. The results of RDA demonstrated that In and Ta were the main factors influencing the photochemistry of *T. media* and *T. mairei*, and their sensitivities to these two environmental factors were different. Therefore, the protection of *Taxus* from potential environmental stresses induced by seasonal changes need to comprehensively consider the tree species and priority of main influencing factors. The seasonal changes disturbed the photosynthetic physiology of *Taxus*; simultaneously, a series of physiological chain reactions occurred to maintain the normal photosynthetic process. As important osmoregulatory substances, the soluble sugar and soluble protein demonstrated almost the same response patterns in the two *Taxus* species throughout the measurements. They increased sharply in spring and midsummer; while the soluble sugar content in both species was also higher in early winter. Previous studies have reported that tree species with higher levels of soluble sugars accumulation could maintain cell turgor and water potential to ensure stomatal opening and stability of photosynthesis [53]. Therefore, the *Taxus* that accumulate more soluble sugars and consume them at a lower rate are presumed to be more tolerant to environmental stress. The soluble proteins are mostly enzymes for various biological processes, which increased in content at the beginning of environmental stresses, and gradually degraded as stresses continued or seasons changed. Interestingly, *T. media* always maintained higher levels of soluble proteins than *T. mairei* in winter. This could be an important clue for the stronger tolerance of *T. media* to the winter environment. Moreover, changes in the photosynthetic physiological states are also due to activity of the protective enzyme system, the increase in which could further increase the Chl fluorescence yield [54,55]. In this study, both *Taxus* species demonstrated similar trends in SOD activity, with no changes in winter but a significant decrease in summer. This suggested that the SOD activity for reactive oxygen scavenging are less important as a photoprotective mechanism for environmental acclimation, which was consistent with the results reported by Verhoeven et al. [7]. However, the POD activity demonstrated a seasonal increase with a different sensitivity in different *Taxus* species, indicating that the POD activity appeared to be the main protective enzyme involved in the protection of the photosynthetic apparatus in *Taxus*. The results of the Pearson correlation analysis and RDA further confirmed the impact of the protective enzyme system on the photochemistry of *Taxus* induced by environmental factors and the potential interspecific differences. Since the Chl fluorescence dynamics and physiological metabolisms of *Taxus* are continuous processes, combined with the complex and varied environmental factors in the field, which makes the results in this study still need the support of large-scale observations for many years, this further demonstrates the diversity of CF-imaging. The subsequent research should focus on the optimization of the combination and analysis mode for the measured data and environmental factors, which is crucial for the surveillance and conservation management of *Taxus*.

## 4. Materials and Methods

### 4.1. Experimental Site and Plant Material

The experimental site is in the germplasm nursery for *Taxus* species located in Nanjing botanical garden Mem. Sun Yat-Sen, Nanjing, China (32°3′ N, 118°49′ E), characterized by a typical subtropical humid monsoon climate with an annual average temperature of 16.2 °C, and an annual average precipitation of 1013 mm. The soil type at the experimental site is yellow-brown earth with a pH of 6.68 and moderate fertility. The twelve-year-old trees of *T. media* and *T. mairei* that were used in this study were planted in the germplasm nursery according to the planting density of 1 m × 1.5 m under the same conditions, such as sunshine and water. Ten individuals with uniform and well-grown conditions were prepared as per *Taxus* species for the subsequent measurements. All the field measurements were carried out on clear, cloudless days between the 10th and 15th of each month from January to December, 2021. Mature and healthy leaves from the third to sixth branch (from top to bottom) per plant were marked for the measurement of Chl *a* fluorescence transient. Then, the leaves were collected for CF-Imaging and measurements of other physiological parameters. The monthly climatic data including the net radiation intensity (In), relative humidity (RH), precipitation amount (P), and mean temperature (Ta) were assessed on the basis of climatic service by Wheat A software (version 1.4.9a) (accessed on 20 August 2022 http://www.wheata.cn) (Table S1).

### 4.2. Measurement of Chlorophyll a Fluorescence Transient

The Chl *a* fluorescence transient was measured with a Handy PEA (Hansatech, Instruments Ltd., Norfolk, UK) on the marked leaves of two *Taxus* species. Before the measurements, all the leaves were dark-adapted for 30 min with leaf clips. Then, the OJIP kinetics of transients were induced with a pulse of saturating red light of 3000 μmol (photon) m^–2^ s^–1^. The fluorescence intensity was recorded from 10 μs to 2 s, and the data were analyzed using the JIP-test [21]. For each *Taxus* species, measurements were repeated four times per month. The introduced basic fluorescence parameters were listed below: Vj, relative variable fluorescence at the J-step; Vi, relative variable fluorescence at the I-step; Sm, standardized area above the fluorescence curve between Fo and Fm is proportional to the pool size of the electron acceptors Q_A_ on the reducing side of PSII; N, the times Q_A_ is reduced while fluorescence reaches its maximal value (number of Q_A_ redox turnovers until Fm is reached); φE_0_, the quantum yield for electron transport from Q_A_^−^ to plastoquinone; φR_0_, the quantum yield for reduction in the end electron acceptors at the PSI acceptor side; ABS/RC, average absorbed photon flux per PSII reaction center (RC); TR_0_/RC, trapping flux leading to Q_A_ reduction per RC; ET_0_/RC, electron transport flux per RC at t = 0; DI_0_/RC, dissipated energy flux per RC at t = 0; ABS/CSm, absorbed photon flux per excited cross section (CS) at t = t_Fm_; TR_0_/CSm, maximum trapped exciton flux per CS at t = t_Fm_; ET_0_/CSm, electron transport flux per CS at t = t_Fm_; DI_0_/CSm, dissipated energy flux per CS at t = t_Fm_; PI_abs_, performance index for energy conservation from photons absorbed by PSII antenna to the reduction of Q_B_; PI_total_, performance index for energy conservation from photons absorbed by PSII antenna to the reduction of PSI acceptors.

### 4.3. Measurement of Chlorophyll Fluorescence-Imaging

Fluorescence images of the leaf surface were obtained using a CF-Imaging system (CF Imager, Technologica, Essex, UK) following the manufacturer’s instructions. Detached leaves of *Taxus* species were dark-adapted for 30 min in a measuring chamber to evaluate the dark-adapted minimum fluorescence (Fo) and dark-adapted maximum fluorescence (Fm). Then, actinic light of 600 μmol (photon) m^−2^ s^−1^ was switched on with saturating pulses of 1800 μmol (photon) m^−2^ s^−1^ repeated every 25 s for steady-state fluorescence (Fs) and light-adapted maximum fluorescence (Fm′); while light-adapted minimum fluorescence (Fo′) was recorded after 3 s of far-red light illumination as the actinic light was switched off. The images and corresponding fluorescence parameters including the maximum quantum efficiency of PSII photochemistry Fv/Fm [Fv/Fm = (Fm − Fo)/Fm], effective quantum yield of PSII photochemistry Fv′/Fm′ [Fv′/Fm′ = (Fm′ − Fo′)/Fm′], non-photochemical quenching NPQ (NPQ = Fm/Fm′ − 1), photochemical quenching coefficient qP [qP = (Fm′ − Fs)/(Fm′ − Fo′)], and actual quantum yield of PSII photochemistry φPSII [φPSII = (Fm′ − Fs)/Fm′] were obtained by the internal software Fluor Imager (version 2.2). For each *Taxus* species, measurements were repeated three times per month.

### 4.4. Measurement of Photosynthetic Pigment Content

The Chl *a*, Chl *b*, total Chl and carotenoid contents in the leaves of *T. media* and *T. mairei* were measured with an 80% acetone extraction according to the method by Wang et al. [20]. The samples (0.1 g) were homogenized in 10 mL of 80% acetone, and homogenates were centrifuged at 4 °C for 15 min (6000× *g*). The supernatants were analyzed by a UV-2102PC/PCS ultraviolet spectrophotometer (UNICO, Shanghai, China). The absorbance was determined at 665, 649, and 470 nm spectrophotometrically. Contents of these pigments were calculated following Lichtenthaler and Buschmann [56].

### 4.5. Measurement of Soluble Protein and Soluble Sugar

Soluble protein content was measured using the Coomassie Brilliant Blue G-250 method described by Bradford [57]. Fresh needles of *Taxus* species (0.5 g) were manually ground and extracted with pre-cooled phosphate buffer (pH 7.0). After centrifugation, the supernatant was mixed with Coomassie Brilliant Blue G-250, and the absorbance of the blue color was read at 595 nm by a spectrophotometer. The amount of soluble protein was calculated with bovine serum albumin as a standard using the following equation: y = 0.007x + 0.0488.

Soluble sugar content was measured with the anthrone-sulfuric acid method [58]. Distilled water was added to the samples (0.1 g) and warmed at 100 °C for 30 min. The samples were extracted two times, each on 10 mL, and the extract was diluted to 25 mL by distilled water. Then, the extract was mixed with anthrone ethyl acetate and 98% sulfuric acid. After heating in a boiling water bath for 1 min, the absorbance of the mixture was read at 625 nm. The amount of soluble sugar was calculated with glucose as a standard using the following equation: y = 0.0037x + 0.0091.

### 4.6. Measurement of SOD and POD Activity

The SOD and POD activity was measured using the method reported by Zu et al. [14]. Fresh needles of *Taxus* species were ground and extracted with a pre-cooled phosphate buffer (pH 7.0). After centrifugation, the supernatant was collected and used for enzyme activities. SOD was assayed by a reaction of the extract mixed with 50 mM phosphate buffer (pH 7.8), 0.1 mM EDTA, 13 mM methionine, 0.02 mM riboflavin, and 0.75 mM nitro blue tetrazolium, which was carried out under illumination at 25 °C for 20 min. Then, the absorbance was measured at 560 nm to determine the SOD activity. The POD was determined by a reaction of the extract mixed with 50 mM phosphate buffer (pH 7.0) and 0.5 mM ascorbic acid. This reaction was started by adding 0.1 mM H_2_O_2_, and POD activity was determined by the rate of guaiacol oxidation at 470 nm.

### 4.7. Statistical Analysis

In this study, the experiments were replicated twice independently. The results were expressed as mean ± SE of at least three biological replicates. The student’s *t*-test was performed using SPSS 19.0 for statistical analysis. Data normality and variance homogeneity were tested with Shapiro and Levene’s tests in SPSS 19.0 prior to the statistical analysis. Pearson correlation analysis was performed to reveal a correlated degree of Chl fluorescence and physiological indicators by using an online tool ChiPlot (accessed on 23 August 2022 https://www.chiplot.online/). A redundancy discriminant analysis (RDA) was performed to reveal the interactions between the environmental factors and measured indicators by using Canoco 5.0 (Microcomputer Power, Ithaca, NY, USA). ChiPlot and GraphPad Prism 8.0 were used to draw the plots.

## 5. Conclusions

In summary, the photosynthetic physiology of *T. media* and *T. mairei* was mainly influenced by In and Ta in the experimental site with seasonal changes. The Fv/Fm-based CF-Imaging technology provided information on the photosynthetic activity and visualized the heterogeneity over the leaf phenotypes, which was expected to be an effective tool to evaluate physiological stress states of *Taxus*. Notably, both *Taxus* species could maintain a stable φPSII under various environmental conditions. This was mainly due to the regulation on the absorption and conversion of light energy by their photosynthetic apparatus, which should be fully considered when determining the damage degree to which *Taxus* was exposed. According to the results of Chl *a* transient, the activity of the electron transport chain in the two *Taxus* species differed to varying degrees in the representative months, which could explain the different values of photochemical parameters for both species growing in similar environmental conditions. Moreover, the diverse energy dissipation approaches were crucial for environmental acclimation. During this period, the osmotic regulation and increase in antioxidant activity were also observed, which contributed to a stable photosynthetic structure and function.

## Figures and Tables

**Table 1:** Seasonal dynamics of chlorophyll fluorescence parameters in *T. media* and *T. mairei*.

	Fv/Fm		Fv′/Fm′		NPQ		qP		φPSII	
	*T.* *media*	*T.* *mairei*	*T.* *media*	*T.* *mairei*	*T.* *media*	*T.* *mairei*	*T.* *media*	*T.* *mairei*	*T.* *media*	*T.* *mairei*
January	0.67 ± 0.03 *	0.60 ± 0.02	0.50 ± 0.05	0.45 ± 0.02	1.22 ± 0.12 *	0.88 ± 0.17	0.86 ± 0.05 *	0.96 ± 0.02	0.42 ± 0.03	0.45 ± 0.01
February	0.81 ± 0.02 *	0.75 ± 0.01	0.56 ± 0.03	0.57 ± 0.03	2.53 ± 0.50 *	1.40 ± 0.22	0.79 ± 0.03	0.75 ± 0.06	0.44 ± 0.01	0.42 ± 0.04
March	0.78 ± 0.01 *	0.70 ± 0.03	0.59 ± 0.04 *	0.48 ± 0.02	1.79 ± 0.30	1.66 ± 0.17	0.80 ± 0.04 *	0.91 ± 0.03	0.47 ± 0.01 *	0.44 ± 0.01
April	0.83 ± 0.02	0.82 ± 0.01	0.61 ± 0.03	0.57 ± 0.02	2.15 ± 0.19	2.60 ± 0.29	0.70 ± 0.04	0.72 ± 0.01	0.42 ± 0.01	0.41 ± 0.02
May	0.84 ± 0.01	0.82 ± 0.01	0.61 ± 0.01 *	0.57 ± 0.02	2.38 ± 0.11	2.46 ± 0.27	0.69 ± 0.01	0.73 ± 0.02	0.42 ± 0.01	0.41 ± 0.01
June	0.83 ± 0.01 *	0.77 ± 0.03	0.62 ± 0.01 *	0.57 ± 0.02	2.21 ± 0.28	1.80 ± 0.59	0.75 ± 0.03	0.74 ± 0.06	0.46 ± 0.02	0.42 ± 0.02
July	0.81 ± 0.01	0.82 ± 0.01	0.62 ± 0.01	0.58 ± 0.04	1.79 ± 0.12 *	2.41 ± 0.23	0.73 ± 0.01	0.71 ± 0.05	0.45 ± 0.01 *	0.41 ± 0.01
August	0.82 ± 0.02	0.79 ± 0.02	0.61 ± 0.01 *	0.56 ± 0.02	1.97 ± 0.41	2.24 ± 0.66	0.77 ± 0.01	0.78 ± 0.06	0.46 ± 0.01 *	0.43 ± 0.02
September	0.81 ± 0.03	0.83 ± 0.01	0.61 ± 0.03	0.58 ± 0.01	1.89 ± 0.14 *	2.61 ± 0.47	0.65 ± 0.05 *	0.77 ± 0.02	0.39 ± 0.04	0.45 ± 0.02
October	0.76 ± 0.02	0.79 ± 0.02	0.59 ± 0.02	0.58 ± 0.01	1.85 ± 0.26 *	2.46 ± 0.28	0.77 ± 0.02	0.80 ± 0.06	0.46 ± 0.01	0.46 ± 0.02
November	0.75 ± 0.01	0.74 ± 0.02	0.60 ± 0.02 *	0.54 ± 0.04	1.57 ± 0.09 *	2.25 ± 0.25	0.76 ± 0.02 *	0.84 ± 0.02	0.46 ± 0.01	0.46 ± 0.03
December	0.71 ± 0.03	0.70 ± 0.05	0.56 ± 0.02	0.55 ± 0.05	1.24 ± 0.16	1.35 ± 0.17	0.81 ± 0.03	0.83 ± 0.03	0.46 ± 0.01	0.45 ± 0.02

Data are shown as mean ± SE (*n* = 3). * indicates significant difference between *T. media* and *T. mairei* in the same month according to Student’s *t*-test (*p* < 0.05).

## Data Availability

The data supporting the findings of this study are available within the article (and its supplementary information files).

## Supplementary Materials

The following supporting information can be downloaded at: https://www.mdpi.com/article/10.3390/plants12142636/s1, Table S1: Monthly climatic data of Nanjing city in 2021; Table S2: Selected JIP-test parameters of *T. media* and *T. mairei* in January, April, July, and October.
