# Supplementary material for: Effects of Seasonal Changes on Chlorophyll Fluorescence and Physiological Characteristics in the Two Taxus Species
Source: Plants (Basel). 2023 Jul 13;12(14):2636. doi: 10.3390/plants12142636 (PMC10384244; doi:10.3390/plants12142636)
Supplement: Supplementary file 1 [file plants-12-02636-s001.zip › plants-2488359-supplementary.pdf]

Table S1 Monthly climatic data of Nanjing city in 2021.

month	mean temperature (Ta) ℃	precipitation (P) mm	relative humidity (RH) %	net radiation intensity (In) net,J/m^2/d
January	4.58	33.02	62.06	9203396.4
February	9.51	35.05	65.05	10904655
March	12.02	76.45	70.6	11135178
April	16.27	46.99	71.05	13222898
May	22.17	225.04	69.33	15584614
June	26.08	77.72	68.18	15637209
July	28.19	385.32	82.56	12594675
August	27.48	199.9	83.74	13387171
September	26.15	38.86	75.27	13656638
October	18.98	120.4	70.04	10583649
November	12.09	23.11	58.37	10024054
December	6.22	3.56	46.71	9123210

Table S2 Selected JIP-test parameters of *T. media* and *T. mairei* in January, April, July, and October.

	January		April		July		October	
	T.media	*T.mairei*	*T.media*	*T.mairei*	*T.media*	*T.mairei*	*T.media*	*T.mairei*
Technical flurescence parameters								
V_j	0.46b	0.55a	0.44b	0.44b	0.44b	0.45b	0.40b	0.44b
V_i	0.77cd	0.80bc	0.79cd	0.75d	0.86a	0.83ab	0.78cd	0.78cd
S_m	38.26a	35.15ab	29.06bc	36.20ab	16.33d	20.21d	28.10c	27.62c
N	41.56bc	49.59a	35.29cd	47.81ab	23.97d	34.16cd	31.59cd	36.97bc
Quantum yields								
φE_0	0.33c	0.24d	0.48ab	0.45ab	0.48ab	0.45b	0.50a	0.47ab
φR_0	0.14b	0.11c	0.18a	0.20a	0.12bc	0.14b	0.19a	0.18a
Specific energy fluxes (per active PSII reaction center)								
ABS/RC	1.79c	2.64a	1.43de	1.63cd	1.72c	2.09b	1.34e	1.61cd
DI_0/RC	0.68b	1.23a	0.22d	0.31cd	0.25d	0.39c	0.22d	0.27d
TR_0/RC	1.11e	1.41bc	1.22de	1.32cd	1.47b	1.70a	1.12e	1.34bcd
ET_0/RC	0.59e	0.63de	0.68cde	0.73bcd	0.83b	0.93a	0.67cde	0.75bc
Phenomenological energy fluxes/activities (per excited cross section CS)								
ABS/CS_m	1060.25c	810.75c	2461.25b	2164.00b	3156.50a	3162.75a	3080.50a	2530.00b
DI_0/CS_m	402.75bc	373.00c	370.25c	408.25bc	466.25b	595.00a	493.50b	429.25bc
TR_0/CS_m	657.50c	437.75c	2091.00b	1755.75b	2690.25a	2567.75a	2587.00a	2100.75b
ET_0/CS_m	349.75d	195.00d	1181.75bc	974.25c	1515.75a	1410.75ab	1540.50a	1196.00bc
Performance indexes (combination of parameters)								
PI_{abs}	1.05e	0.36e	5.24a	3.42cd	4.35b	2.56d	5.82a	4.00bc
PI_{total}	0.78cd	0.30d	3.21ab	2.83ab	1.45c	1.14c	3.45a	2.60b

Data are shown as the average of 4 replicates. Different lowercase letters indicate significant difference between two *Taxus* species in different months according to Duncan's multiple range tests ($P<0.05$).
